# Complexity-based measures inform tai chi’s impact on standing postural control in older adults with peripheral neuropathy

**DOI:** 10.1186/1472-6882-13-87

**Published:** 2013-04-16

**Authors:** Brad Manor, Lewis A Lipsitz, Peter M Wayne, C-K Peng, Li Li

**Affiliations:** 1 Department of Gerontology, Beth Israel Deaconess Medical Center, 110 Francis Street Suite 1B, Boston, MA, USA; 2 Harvard Medical School, Boston, MA, USA; 3 Center for Dynamical Biomarkers and Translational Medicine, National Central University, Chungli, Taiwan; 4 Institute for Aging Research, Hebrew SeniorLife, Roslindale, MA, USA; 5 Osher Center for Integrative Medicine, Brigham and Women’s Hospital and Harvard Medical School, Boston, MA, USA; 6 Division of Interdisciplinary Medicine and Biotechnology and Margret and H. A. Rey Institute for Nonlinear Dynamics in Medicine, Beth Israel Deaconess Medical Center, Boston, MA, USA; 7 Department of Health & Kinesiology, Georgia Southern University, Statesboro, GA, USA

**Keywords:** Tai Chi, Posture, Sway, Balance, Complexity, Intervention, Neuropathy

## Abstract

**Background:**

Tai Chi training enhances physical function and may reduce falls in older adults with and without balance disorders, yet its effect on postural control as quantified by the magnitude or speed of center-of-pressure (COP) excursions beneath the feet is less clear. We hypothesized that COP metrics derived from complex systems theory may better capture the multi-component stimulus that Tai Chi has on the postural control system, as compared with traditional COP measures.

**Methods:**

We performed a secondary analysis of a pilot, non-controlled intervention study that examined the effects of Tai Chi on standing COP dynamics, plantar sensation, and physical function in 25 older adults with peripheral neuropathy. Tai Chi training was based on the Yang style and consisted of three, one-hour group sessions per week for 24 weeks. Standing postural control was assessed with a force platform at baseline, 6, 12, 18, and 24 weeks. The degree of COP complexity, as defined by the presence of fluctuations existing over multiple timescales, was calculated using multiscale entropy analysis. Traditional measures of COP speed and area were also calculated. Foot sole sensation, six-minute walk (6MW) and timed up-and-go (TUG) were also measured at each assessment.

**Results:**

Traditional measures of postural control did not change from baseline. The COP complexity index (mean±SD) increased from baseline (4.1±0.5) to week 6 (4.5±0.4), and from week 6 to week 24 (4.7±0.4) (p=0.02). Increases in COP complexity—from baseline to week 24—correlated with improvements in foot sole sensation (p=0.01), the 6MW (p=0.001) and TUG (p=0.01).

**Conclusions:**

Subjects of the Tai Chi program exhibited increased complexity of standing COP dynamics. These increases were associated with improved plantar sensation and physical function. Although more research is needed, results of this non-controlled pilot study suggest that complexity-based COP measures may inform the study of complex mind-body interventions, like Tai Chi, on postural control in those with peripheral neuropathy or other age-related balance disorders.

## Background

Falls due to poor balance are common and catastrophic in older adults. Lower-extremity somatosensory impairments associated with peripheral neuropathy affect over 20 million U.S. citizens [1] and are a leading cause of poor balance in this population [2]. As the U.S. healthcare cost of fall-related injuries—which was 20 billion in 2000—is projected to escalate to 54 billion by 2020 [3], determination and implementation of interventions that alleviate the consequences of peripheral neuropathy on balance is critical.

Tai Chi, also known as Tai Chi Chuan or Taijiquan, stems from the Chinese martial and healing arts. As a therapeutic tool, Tai Chi is best viewed as a multi-component exercise including detailed regimens of physical movement, breathing techniques and cognitive tools (both visualization and focused internal awareness) [4-6]. Tai Chi is widely purported to improve balance. Systematic reviews suggest Tai Chi practice can directly reduce fall risk [7-9] and/or positively impact factors associated with falling including the fear of falling [9,10], clinical measures of balance [11-13], musculoskeletal strength [12,13] and flexibility [11,12,14]. Several studies have specifically reported that Tai Chi training may improve clinical measures of balance and gait in those with peripheral neuropathy [15,16].

The effects of Tai Chi on postural control in older adults with and without peripheral neuropathy, however, are less clear. Whereas some studies report reductions in the average speed or magnitude of center-of-pressure (COP) fluctuations beneath the feet when standing [17,18], others report no change [15,19-21] or even increases [9] in these parameters. These inconsistencies may stem from inherent limitations of traditional COP metrics, which often fail to capture important dynamic information contained within the signal. There is a growing appreciation that COP dynamics measured beneath the feet are highly “complex;” i.e., they contain non-random fluctuations that exist across multiple temporal and spatial scales [22,23]. Physiologic complexity arises from the rich network of feedback loops that are integrated with subcortical, cortical and peripheral motor circuitry to regulate one’s posture [24]. As such, the degree of physiologic complexity contained within the dynamics of a system under basal or “free-running” conditions is believed to fundamentally relate to the capacity of that system to adapt to the exigencies of everyday life [25]. Current research indicates that the complexity of COP dynamics may be uncorrelated with traditional COP parameters [26,27], is closely linked with frailty [27] and falls [28], and is diminished in those with peripheral neuropathy [26].

Tai Chi is an inherently complex intervention that targets multiple physiological and biomechanical elements of the postural control system. We and others have therefore proposed that the impact of Tai Chi on postural control may be better characterized by quantifying its effects on the degree of complexity associated with system output (i.e., COP dynamics). To date, we are not aware of any attempt to apply complexity measures of COP data to evaluate the impact of Tai Chi on postural control.

In a previous study we reported the impact of a 24-week Tai Chi intervention in older adults with peripheral neuropathy [15]. Despite significant improvements in leg strength, mobility, and even the perception of light touch on the foot soles, no changes were observed in standing postural control as quantified by traditional COP metrics (i.e., average speed and magnitude). These traditional metrics, however, may not have been sensitive to potential changes in the complexity of COP dynamics following Tai Chi training. In recent years, we have developed a measure, termed multiscale entropy, which can be applied to capture COP complexity [22]. This metric is sensitive to the change of sensory information. For example, older adults with chronic visual and/or sensory impairments have lower COP complexity when standing quietly (26). Moreover, we also observed that COP complexity values acutely increase when foot sole somatosensory feedback is enhanced by applying sub-threshold mechanical vibrations to the foot soles [28]. In this present study, we performed a secondary analysis of the same data to explore the value of multiscale entropy-derived COP complexity, as well as the relationship between COP complexity and function. We hypothesized that as compared to traditional metrics, the degree of complexity associated with COP dynamics would better capture the multi-component stimulus provided by Tai Chi training.

## Methods

### Trial design

We tested our hypotheses by performing a secondary analysis of a previously reported non-controlled and non-randomized pilot study [15].

### Subjects

Subjects were recruited into the original study from the community via advertisements and educational seminars between 2005 and 2008. All subjects provided written informed consent of all study procedures, which were approved by the Louisiana State University Institutional Review Board. Inclusion criteria were physician-diagnosed peripheral neuropathy and the ability to stand unassisted with eyes-closed. Exclusion criteria were foot ulceration, any other movement disorder, or any uncontrolled cardiovascular, respiratory, or metabolic disorder. We screened 38 individuals, of which 28 were eligible and agreed to participate in 24 consecutive weeks of Tai Chi training classes offered through a community outreach program at Louisiana State University. Three subjects did not complete the training program due to non-medical personal reasons and were excluded from analysis.

### Tai chi training protocol

Subjects were asked to complete three, one-hour group Tai Chi training sessions per week for 24 weeks. Group sessions were limited to subjects within the study and contained a maximum of 15 subjects (two identical sessions were offered each day). They were taught by a single instructor with 40 years of experience, including ample research experience with balance impaired populations. Training focused on ten movements selected from the 24-form Yang Style Tai Chi, similar to that described by Wolf et al. [9], and emphasized slow intentional movement, transfer of body weight between the lower-extremities, and controlled breathing.

### Functional assessment and analysis

Postural control, foot sole sensation, leg strength, functional capacity and mobility were assessed within the Biomechanics Laboratory at Louisiana State University at baseline and every six weeks throughout the training program.

#### Postural control

Postural control was assessed by recording COP dynamics at 50 Hz during three, 30 sec trials of eyes-closed standing (arms at side, heels 5 cm apart, and feet abducted 10deg) on a stationary force platform (AMTI, Watertown, MA).

For this secondary analysis, we computed a COP complexity index using multi-scale entropy analysis [22]. This metric estimates the degree of “information content” of a physiological signal. With respect to standing postural control, this metric is believed to reflect the combined influences of the numerous inputs (i.e., sensory, sensory integration, and motor elements) that interact with one another nonlinearly to regulate the body’s postural sway (i.e., COP) over time. Specifically, the metric characterizes the degree of irregularity within the signal over multiple temporal scales, such that greater multi-scale irregularity reflects higher information content, or complexity. It is suitable for relatively short, nonstationary time-series [23,29] and has high test-retest reliability in community-dwelling older adults [27]. It also offers distinct advantage over traditional entropy metrics, which are limited to the estimation of regularity on a single time scale and thus, have no straightforward correspondence to physiologic complexity [22].

Reliable entropy analysis requires the occurrence of multiple repetitions of a given dynamical pattern. Therefore, relatively low frequency non-stationarities were filtered using Empirical Mode Decomposition [29,30]. The EMD method decomposes a signal into *n* "intrinsic mode functions," where each function is characterized by a dominant frequency equal to the sampling frequency divided by 2^*n*+1^. Here, we restricted our analysis to intrinsic mode functions 1–3, thus filtering dynamical information on time scales larger than 320 ms (i.e., 2^3+1^/50 sec, where 1/50 sec is the sampling interval).

To ensure that COP dynamics across the bandwidth of interest were distinguishable from noise, we recorded the COP fluctuations of a 75 Kg mass and compared its power in each principle direction to all acquired COP time-series. In all cases, the signal-to-noise ratios in the anterioposterior (AP) and mediolateral (ML) directions were greater than ten and less than one, respectively. For this reason, we only computed the complexity index (see below) from AP COP dynamics.

The complexity index was calculated for each filtered AP COP time-series using a three step process. First, the time-series was “coarse-grained” to produce multiple time-series that each capture system dynamics on a given time scale. The coarse-grained time-series for time scale *n* is the sequence of mean center-of-pressure values produced by dividing the original time-series into non-overlapping windows with *n* data points, and then calculating the mean value for each window. As entropy analysis (see second step) is a statistical measure, the length of time-series must be substantially longer than the time-scale of interest to ensure sufficient samples for the analysis [31]. As such, each time-series was coarse-grained into scales 1 (i.e., 1500 data points) to 5 (i.e., 300 data points).

Second, the degree of irregularity associated with each coarse-grained time-series was calculated using sample entropy, such that greater entropy reflects greater irregularity at that time-scale [31]. This conditional probability metric quantifies the likelihood that if a vector with *m* data points matches a template of the same length, within a tolerance *r*, the vector and template will still match when their length increases from *m* to *m* + 1 data points. Here, we used *m*=2 and *r*=15% of the standard deviation of the original signal [22,31,32].

Third, the COP complexity index was calculated by plotting the sample entropy of each coarse-grained time-series as a function of time scale, and then calculating the area under the resultant curve [28]. As such, time-series with greater irregularity over multiple temporal scales have more area beneath the curve and higher complexity.

In addition to the COP complexity index, the traditional balance measure of average COP speed was computed by dividing total path length by trial duration. COP area was calculated by computing the area of an ellipse enclosing 95% of the COP signal.

#### Foot sole sensation

The ability to perceive the 5.07 gauge Semmes–Weinstein monofilament (North Coast Medical Inc.) was assessed at the heel, mid-sole, bases of first and fifth metatarsals, and hallux. Each site was assessed three times, and intact sensation was defined by two or more correct responses [33].

#### Leg strength

Knee extensor and flexor peak torque was measured with a Biodex dynamometer (Biodex Medical, Shirley, NY). Following warm-up, five maximal, reciprocal knee extension and flexion movements were completed at 60 degrees/sec with 10 seconds of rest between trials. Visual feedback and verbal encouragement were provided. Peak torque was determined by averaging maximal values from the three best trials of each movement direction.

#### Functional capacity and mobility

Functional capacity was determined by the distanced covered in the 6MW, which was completed on a 30 m course along an indoor hallway. Functional mobility was determined by TUG test performance, as measured by the time taken to rise from a chair, walk around a cone 3m away, and sit back down in the chair. The average time of two separate trials was used in the analysis.

#### Statistical analysis

Statistical analyses were performed using JMP software (SAS Institute, Cary, NC). All 25 subjects were included in the analysis and descriptive statistics were used to summarize each variable. The effects of Tai Chi exercise on COP complexity, area and speed were analyzed with one-way, repeated-measure ANCOVAs with testing session (baseline, 6, 12, 18, and 24wk) as the within-subject factor. Covariates included subject characteristics known to influence postural control (i.e., age, sex, and BMI), foot sole sensation at baseline (i.e., the number of sites with intact sensation) and the number of completed practice sessions. Tukey’s post-hoc testing was used to compare factor means of significant models. As a secondary analysis, the relationship between changes in COP metrics and changes in foot sole sensation, leg strength (i.e., knee extensor and flexor peak torque), functional capacity (i.e., 6MW) and mobility (i.e., TUG) were calculated. Spearman or Pearson product correlations were used for ordinal and continuous data, respectively. Linear regression analyses were also performed to examine each relationship while controlling for age, sex and BMI. Significance level was set at α=0.05 for all analyses.

## Results

### Subject characteristics

Twenty five subjects (17 women and 8 men, age=71.11±12.8 years, body mass=76.0±18.1 kg, height=168.2±9.0 cm, time since PN diagnosis=6.3±4.2 years) completed 69.0±5.1 out of 72 sessions (range=58-72 sessions). Eight subjects were diagnosed with type 2 diabetes mellitus. The remaining subjects presented with idiopathic PN.

### The effect of tai chi on foot sole sensation, leg strength and physical function

As reported previously, foot sole sensation, leg strength and physical function increased from baseline to week 24.^15^ Briefly, subjects exhibited heightened foot sole sensation, as defined by the number of five tested foot sole sites with intact sensation, from 2.8±1.5 to 3.8±1.3 sites (p=0.003). Knee extensor and flexor peak torque rose from 83.5±38.1 to 91.8±38.9 Nm (p=0.009) and from 43.8±14.9 to 49.8±17.3 Nm (p=0.01), respectively. Distance walked in the 6MW increased from 407.2±57.8 to 454.8±63.0 m (p<0.001) and the time needed to complete the TUG decreased from 10.2±2.1 to 8.9±1.6 sec (p<0.001).

### The effect of tai chi on standing postural control

**Figure 1:**
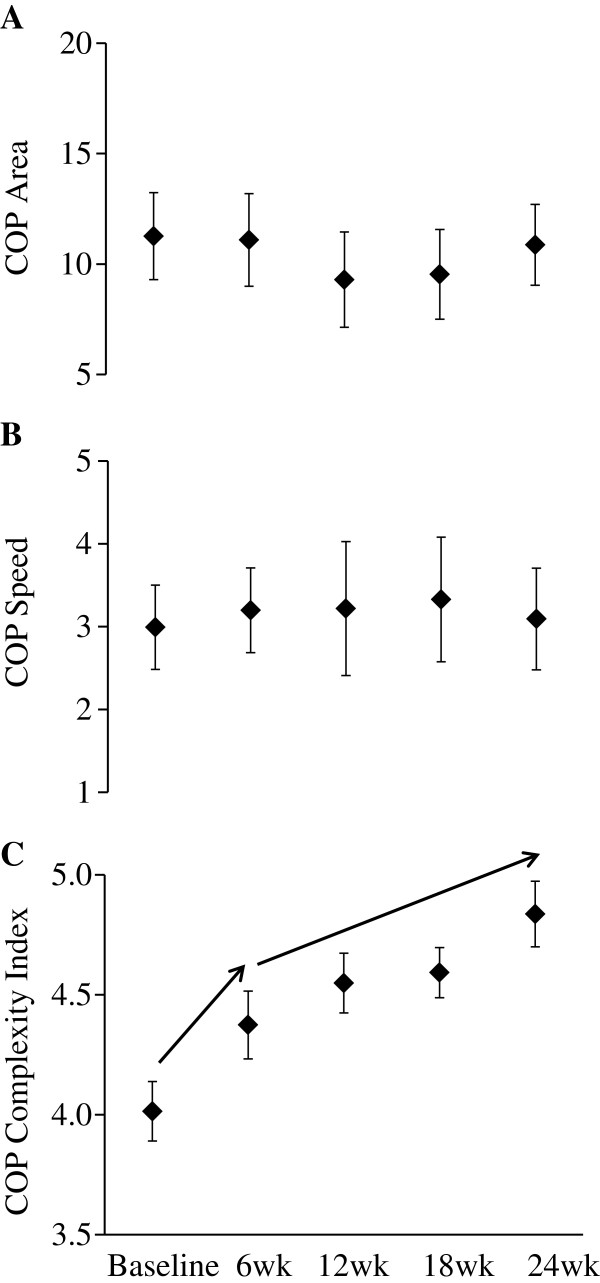
**Standing postural control in older adults with peripheral neuropathy over the course of a 24 week Tai Chi training program. **The Tai Chi training intervention was not associated in significant changes in center-of-pressure (COP) area (**A**) or speed (**B**). On the other hand, subjects demonstrated significant (p<0.05, arrows) increases in COP complexity (**C**) from baseline to week 6, and again from week 6 to week 24. Error bars reflect standard error from the mean.

No changes in COP area or speed were observed during the intervention period. On the other hand, COP complexity increased (F_4,22_=3.19, p=0.02) (Figure 1). Post-hoc testing revealed significant increases between baseline and week six, and between week six and week 24. Observed changes were independent of age, sex, BMI and the number of testing sessions completed.

### Relationships between postural control, foot sole sensation and physical function

**Figure 2:**
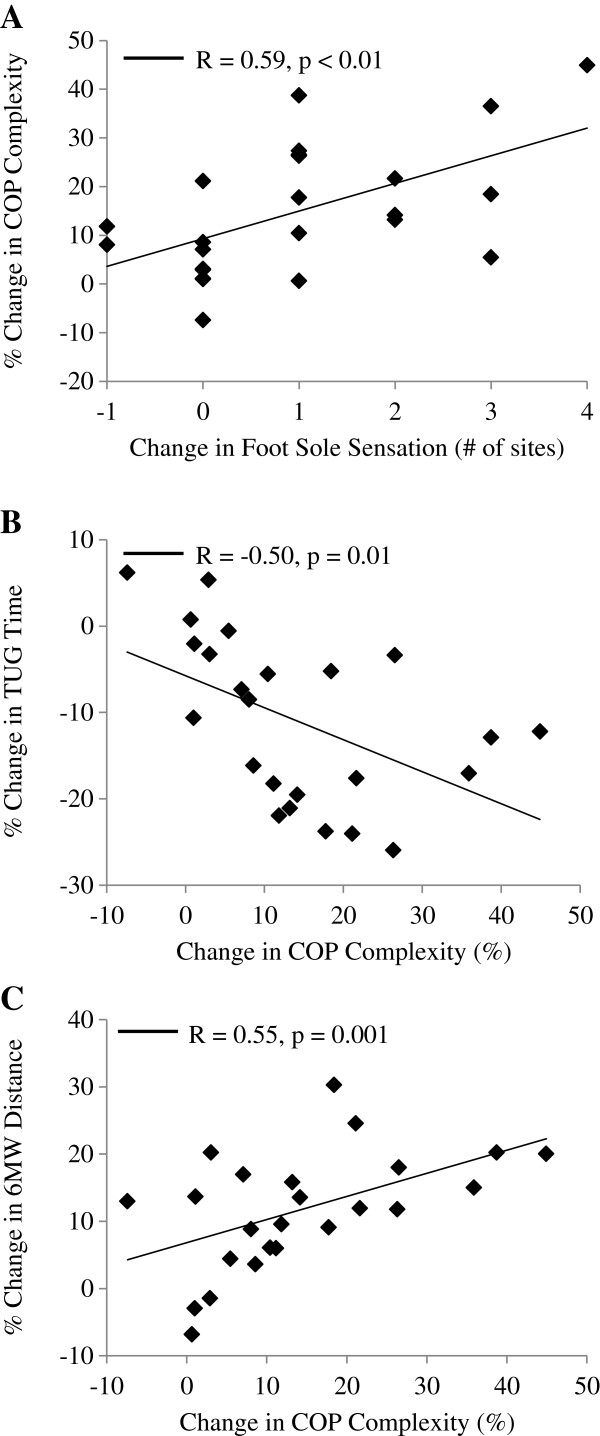
**Relationships between changes in foot sole sensation**, **center**-**of**-**pressure **(**COP**) **complexity**, **and physical function following 24 weeks of Tai Chi training in older adults with peripheral neuropathy. **Improved foot sole sensation, defined as the change in the number of five tested foot sole sites on which the subject could perceive the 5.07 gauge monofilament, correlated with the percent change in the degree of COP complexity during quiet standing with closed eyes (**A**). The percent increase in COP complexity correlated with the percent decrease in the time needed to complete the timed up-and-go test (TUG) (**B**), and the percent increase in the distance covered in the 6 minute walk (6MW) test (**C**). The correlation between changes in traditional COP parameters (area and speed) and physical function (not pictured) were not significant.

Correlation analyses revealed that percent increases in COP complexity, from baseline to week 24, were associated with improvement in foot sole sensation (i.e., the change in the number of five tested foot sole sites on which subjects were able to perceive 10 g of pressure, R=0.59, p<0.01), as well as the percent improvement in functional capacity (i.e., 6MW distance, R=0.55, p=0.01) and mobility (i.e., TUG time, R=-0.50, p=0.01) (Figure 2). Regression models indicated that each of these relationships were independent of age, sex, and BMI (p≤0.01). The observed increase in COP complexity was not related with improvements in knee extensor or flexor peak torque. No significant relationships were observed between changes in traditional COP measures (speed and area) and changes in foot sole sensation, leg strength, functional capacity or mobility.

## Discussion

To date, the predominant paradigm of medical research has been a reductionist one—i.e. complex physiologic systems are simplified into their component parts and evaluated for their relevance to the overall physiological function or behavior in question [34,35]. Behaviors produced by healthy physiologic systems, however, rely upon nonlinear interactions among numerous feedback loops and regulatory processes that operate over multiple temporal and spatial scales [24,36]. Holistic behavior of the system, therefore, may be distinct from the behavior of its component parts [37]. As such, tools derived from complex systems biology that capture the characteristics of integrated, multi-scale control (i.e., physiologic complexity) may be better suited to study human behavior in health and disease, and to evaluate the physiological impact of Tai Chi and other multi-component mind-body exercise interventions.

Biological aging and disease have each been associated with a loss of complexity in the dynamics of numerous physiologic systems [24,36], including the postural control system [26-28]. Few reports, however, have studied the potential to restore physiologic complexity in these vulnerable populations. In the present study, older adults with peripheral neuropathy that completed a Tai Chi training program did not exhibit changes in the speed or magnitude of COP dynamics averaged over 30 seconds of standing. The degree of COP complexity, on the other hand, increased after only six weeks. Increased COP complexity was correlated with both heightened foot sole sensation and improved physical function. Together, these observations suggest that Tai Chi may restore balance in older adults with somatosensory loss by targeting the complex dynamics of postural control.

The results of the current study may help explain mixed reports of Tai Chi’s effects on standing postural control [9,15,17-19]. For example, Wolf et al. [9] reported that although a 15-week Tai Chi intervention reduced the risk of injurious falls and decreased fear of falling in older adults, it did not change, or even increased average measures of COP magnitude in older adults. Similarly, whereas Tsang and colleagues reported that Tai Chi improves the ability to step [38] and shift one’s weight [39], it appears to have little or no effects of static postural control when standing quietly with eyes open [20,21]. In the current study, despite improved mobility [15] after the training program, no changes were observed in the average speed or magnitude of COP fluctuations over the intervention period. Yet, significant increases were observed in COP complexity. Together, these observations suggest that COP complexity captures unique information regarding the postural control system. Furthermore, as compared to traditional metrics, complexity-based metrics may be more discriminating in characterizing the impact of Tai Chi and other multi-component mind-body interventions focused on the rehabilitation of postural control and balance.

The loss of physiologic complexity that often accompanies biological aging and disease is believed to stem from diminished input to the system (i.e., feedback) and/or a breakdown in the underlying networks within the system that regulate behavior over time [24,25,36]. Older adults with relatively low COP complexity associated with the dynamics of quiet standing are more likely to be frail [27], have a history of falls [28], and exhibit reduced capacity to maintain posture during a cognitive stressor [26]. In a study of 453 community-dwelling older adults, we reported that those with chronic impairment in foot sole pressure sensation exhibit less COP complexity when standing quietly as compared to their age-matched counterparts with intact sensation [26]. The current observation that older adults with PN that completed a Tai Chi training program demonstrated increased COP complexity, in concert with improved plantar sensation, suggests that restoration of the multi-scale regulation of standing postural control may have stemmed in part from heightened feedback related to foot sole sensation. As other studies have also reported improved foot sole sensation [16] and increased peripheral nerve conduction properties [40] following Tai Chi training in older adults, future studies are thus needed to determine the mechanisms through which improved sensation may impact the complex control of standing posture.

There are a number of limitations in this pilot study. First, as Tai Chi was not compared to another intervention or standard care, we were unable to definitively determine if the changes we observed in COP complexity, plantar sensation, or function were due to Tai Chi specifically or to a non-specific group effect. Moreover, we were unable to delineate the specific component(s) of the intervention that may have contributed to the observed improvements, which is indeed a major challenge in mind-body research [5]. Significant correlations between increases in COP complexity and improvements in physical function over the study duration, however, suggests that observed changes were in fact due to an intervention effect rather than random variation in the complexity measure over time. Still, larger-scale controlled trials are needed to validate the observations of this relatively small pilot study. While the relationship between increased COP complexity and improved somatosensation is intriguing, somatosensation was based only upon the ability to perceive 10 g of pressure on the foot sole in a non-weight-bearing position. It is therefore unknown if improved somatosensation translated into improved feedback to the postural control system when standing. Finally, multiscale entropy is just one of many metrics that can be used to quantify physiologic complexity. With respect to postural control, this metric is believed to estimate the combined influences of all sensory, sensory integration, and motor elements involved in the complex control of postural sway over time. Future research is needed however, to 1) further compare the results of carefully-designed mechanistic studies of COP complexity with clinical outcomes, and 2) delineate Tai Chi-related changes to the specific control elements mediating increases in COP complexity within this cohort and other aging populations.

## Conclusions

Despite no change in traditional COP parameters of standing postural control, older adults with PN experienced increased COP complexity after completing only six weeks of a Tai Chi training program. As these increases were linked to improved plantar sensation and physical function, this preliminary evidence suggests that metrics targeting the complex dynamics of human postural control may inform the impact of multi-component mind-body interventions in older adults with PN or other age-related balance disorders.

## Competing interests

The authors report no competing interests.

## Authors’ contributions

BM contributed to the design of the study, acquisition, analysis and interpretation of data, and preparation of the manuscript. LAL and PW contributed to interpretation of data and preparation of the manuscript. CKP contributed to data analysis. LL conceived the study and oversaw its conduct, and contributed to interpretation of data and preparation of the manuscript. All authors read and approved the final manuscript.

## Pre-publication history

The pre-publication history for this paper can be accessed here:

http://www.biomedcentral.com/1472-6882/13/87/prepub
